# Development and validation of digital resilience scale for primary and secondary school students

**DOI:** 10.1186/s40359-025-03739-0

**Published:** 2025-11-25

**Authors:** Qianqian Pan, Min Lan, Cheng Yong Tan, Sisi Tao, Qianru Liang, Nancy Law

**Affiliations:** 1 Centre for Research in Pedagogy and Practice, National Institute of Education, Nanyang Technological University, Singapore, Singapore; 2 Zhejiang Key Laboratory of Intelligent Education Technology and Application, Zhejiang Normal University, Jinhua, 321004 Zhejiang China; 3 Faculty of Education, The University of Hong Kong, Pokfulam, Hong Kong China; 4 Department of Early Childhood Education, The Education University of Hong Kong, Ting Kok, Hong Kong China; 5 Guangdong Institute of Smart Education, Jinan University, Guangzhou, China; 6 Institute of High-Quality Education Development, Zhejiang Normal University (Key Cultivation Research Institute Centre of Philosophy and Social Sciences of Zhejiang Province), Jinhua, China

**Keywords:** Digital resilience, Validation, Measurement invariance, Scale development, Primary school students, Secondary school students

## Abstract

**Background:**

With increasing digital engagement, children and adolescents face both opportunities and risks online. While exposure to online risks does not always lead to harm, digital resilience (DR)—the ability to cope with and recover from negative online experiences—mitigates potential adverse effects. Despite its importance, validated tools to assess DR in young populations are scarce. We developed and validated a multidimensional Digital Resilience Scale that assesses coping strategies, bouncing back, and personal growth following online risk exposure.

**Methods:**

Guided by the UK Council for Internet Safety (UKCIS) Digital Resilience Framework (DRF), we validated the scale with 2,014 primary and 6,014 secondary school students in Hong Kong. The instrument comprises two subscales: (1) Coping Strategies (productive coping, reference to others, non-productive coping) across three common online risks (unwanted messages, personal-information leakage, cyberbullying); and (2) Recovery (bouncing back, growth). We conducted confirmatory factor analyses (CFAs), tested measurement invariance across gender and school level, and examined external validity with digital literacy (DL) and well-being.

**Results:**

CFAs supported a five-factor structure (three coping factors, bouncing back, growth) with good fit (CFI = 0.967–1; RMSEA = 0.00 – 0.046). Subscales showed high reliability (ω = 0.764 **–** 0.910). Measurement invariance across gender and school level was supported. DR correlated positively with DL (*r* = 0.036 – 0.254) and well-being (*r* = 0.042 – 0.659), supporting external validity.

**Conclusions:**

The Digital Resilience Scale provides a reliable, multidimensional measure of students’ capacity to navigate and recover from online risks. It offers a practical tool for educators, researchers, and policymakers to assess DR and inform targeted interventions. Future research should evaluate cross-cultural generalizability and longitudinal sensitivity.

**Supplementary Information:**

The online version contains supplementary material available at 10.1186/s40359-025-03739-0.

## Introduction

In today’s digital landscape, children and adolescents encounter many online opportunities that enrich both their educational pursuits and personal lives. These opportunities necessitate the development of digital skills, which can in turn promote positive aspects of their growth, such as self-acceptance and personal development [1]. However, the internet also poses significant risks, including exposure to online hate, cyberbullying, and sexual solicitations, which can adversely impact their well-being [2–5]. Importantly, exposure to such online risks does not always result in negative outcomes [6]. This variability highlights individuals who remain resilient in the face of adverse online experiences and underscores the protective role of digital resilience (DR) [6–8].

Resilience is commonly conceptualized as a process of positive adaptation in the face of adversity, arising from the interplay of risk exposure with promotive assets (e.g., coping skills) and ecological resources [9–11]. Classic accounts emphasize two key points: (a) risk exposure is necessary for resilience to be expressed and (b) adaptation is context- and task-specific; recovery may lead to growth when individuals and contexts mobilize appropriate resources [12]. In the context of digital ecologies, digital resilience denotes a youth’s capacity—when faced with online adversity—to (a) recognise and appraise digital risks, (b) mobilise context-appropriate coping responses, and (c) recover and sometimes grow following such experiences [13–15].

To clarify the concepts central to this study, we distinguish between three constructs: general resilience, digital resilience (DR), and digital literacy (DL). General resilience is the process of positive adaptation in the face of adversity, arising from the interplay of risk exposure with promotive assets (e.g., coping skills) and ecological resources. Digital resilience is a risk-activated, domain-specific process that concerns how young people respond when things go wrong online—recognising and appraising digital risks, selecting and enacting coping strategies, bouncing back, and experiencing post-adversity growth. Because resilience is domain- and risk-specific, general resilience scores cannot be assumed to index DR; purpose-built measures tailored to digital risk experiences are required. Digital literacy refers to the knowledge, skills, and dispositions that enable effective and safe use of digital technologies (e.g., information evaluation, technical/operational skills, safety practices). DL can reduce the likelihood or severity of harm and may act as an antecedent, correlate, or moderator of DR; however, it is not a stress-response process and can be present without exposure to—or recovery from—online adversity [14, 16, 17].Despite growing interest, DR measurement remains underdeveloped. A recent review reports heavy reliance on instruments adapted from adjacent constructs, few purpose-built DR scales, and limited standardization [14, 16]. Complementary conceptual work highlights DR’s multidimensionality and the absence of validated tools spanning technical, operational, and human dimensions [17]. These gaps motivate the present study: to develop psychometrically robust, developmentally appropriate DR measures for children and adolescents that can advance research, inform practice, and support intervention evaluation.

To address these gaps, we develop a multidimensional DR measure grounded in resilience theory [9–11] that instrument is designed to evaluate participants’ behavioural responses to specific digital challenges (i.e., coping strategies) and their ability to recover after exposure to online risks. This includes assessing both their capacity to “bounce back” and their growth following negative experiences. By implementing this tool, the study seeks to enhance our understanding of DR in children and adolescents, providing a scientific foundation for future interventions aimed at fostering mental health and well-being in the digital context.

## Literature review

### Multidimensional construct of digital resilience

Contemporary resilience theory does not conceive of resilience as a unitary attribute, but rather as a dynamic process involving multiple interacting components that unfold over time and across different contexts (e.g., individual, relational, and ecological). Classic and recent syntheses emphasize three points: (1) heterogeneity in responses to adversity (2), the interplay of risk and protective processes, and (3) the need to consider multiple levels of analysis when operationalizing resilience [9, 11, 18]. Thus, resilience is “not a one-dimensional, dichotomous attribute,” and overly generalized, single-facet approaches are discouraged in favor of multidimensional models and interventions. When translated to the digital environment, both conceptual and empirical work can show that young people’s adaptation to online risks comprises two related but separable facets: (1) how they manage an incident (e.g., problem-focused, communicative, or avoidant coping strategies) and (2) how they recover and grow from the experience [19].

Consequently, scholars have proposed a multidimensional construct of digital resilience, and there has been limited empirical evidence collected to evaluate this multidimensional structure. The United Kingdom Council for Internet Safety (UKCIS) has developed a comprehensive framework that outlines the components of digital resilience [13]. This Digital Resilience Framework (DRF) sees digital resilience as a dynamic personal asset that develops through active engagement with appropriate online opportunities and challenges, rather than through avoidance and safety behaviors. The framework encompasses four key areas: understanding when individuals are at risk, knowing how to seek help when needed, learning from past experiences to inform future decisions, and receiving the necessary support to recover and continue to engage with digital opportunities. In accordance with this framework, UKCIS offers a practical and user-friendly self-assessment checklist for institutions to evaluate their efforts to build resilience. At this time, no measurement tools have been developed for measuring individuals’ digital resilience.

In the context of schooling, Sun and colleagues [14] proposed a conceptual framework that defines DR as a capability and a dynamic cyclical process, which enables individuals to change their behavioral performance and psychological functioning when faced with various digital technology-related threats in school settings. Similar to DRF, this framework includes understanding online threats, knowing solutions, acquiring knowledge and skills to adapt future choices, recovering from stress by returning to normal activities, and moving forward with increased self-efficacy. The framework recognises that online risks serve as both antecedents and consequences of digital resilience, which in turn influences students’ behavioral performance (e.g., learning behavior and achievement) and psychological functioning (e.g., social and emotional aspects).

Similarly, Hammond and colleagues [15] describe DR as a multi-stage concept that includes learning about online risks, recognising online risks, managing (also known as using coping strategies, including ignoring and disengaging), and recovering (including acceptance and growth). Their framework adopts a social-ecological perspective, allowing for the exploration of how individual, home, community, and societal levels collectively influence and support children to thrive online.

Across these frameworks, the UKCIS framework provides a more general approach, Sun et al. [14] view DR as a cyclical rather than linear process, while Hammond et al. [15] place greater emphasis on the social context and factors that facilitate children’s development of DR. However, aligning closely with the broader definition of resilience, DR is consistently conceptualized as a multidimensional construct rather than a unidimensional one. DR encompasses the ability to manage online risks, recover from challenges and grow from these experiences, while recognising that online risks will be the precursors.

### Measurement of digital resilience

Rigorous measurement of DR is essential for advancing our understanding of this critical concept and developing targeted interventions that help young people navigate their digital lives, promoting overall well-being. However, there is currently no consensus on the operational definition of DR which has impacted efforts to standardize its measurement. For instance, some researchers defined DR as the ability to bounce back from adversity (e.g., [19]). In other studies, DR was conceptualized as an outcome based on the reported level of harm from negative online experiences, with individuals reporting less harm viewed as more resilient [8, 20]. Furthermore, some studies focused on coping strategies as indicators of digital resilience, with three main types identified. Individuals who engage in communicative coping (e.g., seeking support from parents or friends) and proactive coping (i.e., actively addressing problems) tend to exhibit higher levels of resilience compared to those relying on passive coping (i.e., ignoring problems) [6, 21].

As mentioned above, in recent years, the concept of DR has gained increasing recognition as a multidimensional construct that is aligned with the conceptualisation of general resilience. However, the prevailing measures of DR continue to conceptualize it in a unidimensional manner, potentially impeding a comprehensive understanding of this critical construct. Unidimensional indices fail to differentiate between distinct phases of adaptation (coping vs. recovery/growth), thereby obscuring theoretically meaningful variation and limiting the diagnostic utility of such indices for intervention design. Consequently, there is a growing need for research to develop measurement that can adequately assess DR as a multidimensional construct. This approach is expected to yield a more comprehensive understanding of DR and support the development of interventions designed to cultivate resilience in students.

Despite the mounting interest, extant DR measures exhibit other deficiencies. First, although resilience is context- and content-specific [9, 10, 22], many digital resilience instruments adapt general resilience or safety/literacy items and rarely embed items in concrete risk scenarios or model scenario/testlet dependence. This approach risks construct–measure mismatch and biased parameters [16, 17, 23]. Second, meaningful comparisons across gender and developmental stage require formal tests of measurement invariance [24]. However, these are seldom reported in DR studies. The present study addresses these shortcomings by employing scenario-anchored items and a two-tier item-factor model to account for testlet dependence and conducting multi-group invariance analyses (configural, metric, scalar) across gender and school level.

### Relationships between digital resilience, digital literacy, and well-being

Research consistently suggests that digital resilience acts as a protective factor for young people’s well-being, particularly when they encounter online risks. Individuals with higher levels of digital resilience are better equipped to cope with online threats and recover from these experiences [5, 8, 25]. This capacity to effectively manage and rebound from adverse online situations plays a crucial role in safeguarding their emotional and psychological well-being.

In addition, digital literacy (DL) is widely recognized as a fundamental skill in the 21st century, encompassing the knowledge, abilities, and attitudes necessary for effective navigation in a digital world [26]. Children who are more digitally literate tend to exhibit greater resilience in the face of online adversities [21]. Studies have shown that young people with higher levels of digital skills are more adept at coping with online risks and are better able to avoid or mitigate feelings of harm resulting from these negative experiences compared to those with lower digital literacy [5, 27–29].

Therefore, DL may serve as a critical foundation for fostering DR in students. Both DL and DR are likely to have positive associations with individual well-being, as they enable young people to navigate the digital world more confidently and recover from potential setbacks with greater ease [5].

### Current study

The present study is grounded in the theoretical framework of developmental resilience theory [9, 30], and the DR is operationalized in the context of schooling using the UKCIS Digital Resilience Framework (DRF) [13]. The DRF is complemented by UNESCO’s Digital Kids Asia-Pacific (DKAP) framework, which is utilized to ensure regional relevance [31]. The decision to utilize DRF was not solely influenced by its prevalence in practical applications; it was also due to its provision of an action-oriented, age-appropriate scaffold that encompasses the entire online risk cycle (i.e., the process of understanding risks, identifying sources of assistance, acquiring knowledge from experience, and recuperating to maintain engagement). This theory-consistent coverage directly maps onto our two-facet operationalization—coping during incidents (productive, reference-to-others, non-productive) and recovery after incidents (bouncing back, growth)—and supports content-valid item generation via authentic school-age scenarios (unwanted messages, information leakage, cyberbullying). This specification is employed to assess a five-factor model, as well as to examine reliability, measurement invariance, and external validity with DL and well-being.

The primary objectives of this study are to examine the psychometric properties of the newly developed scale. Specifically, the research aims to answer the following questions:

- RQ1: What are the relationships among the three aspects of digital resilience (coping strategies, bouncing back, and growth)?
- RQ2: What are the psychometric properties of this digital resilience scale, including its validity and reliability and measurement invariance across gender and age groups (primary vs. secondary school students)?
- RQ3: What are the relationships between digital resilience and digital literacy, and between digital resilience and well-being as evidence of external validity?

## Method

### Scale development

Our conceptualization is grounded in developmental resilience theory, which defines resilience as positive adaptation in the face of adversity and treats it as a dynamic process shaped by risks, promotive assets, and contextual resources across ecological levels. To specify how these processes manifest in digital ecologies, we operationalize DR using the UKCIS Digital Resilience Framework (DRF) [13], which articulates action-oriented pillars—understanding risks, knowing where to seek help, learning from experience, and recovering to sustain participation—observable in children’s and adolescents’ online lives. The Digital Resilience Scale (see supplementary materials for details) was developed based on the DRF and further informed by UNESCO’s Digital Kids Asia-Pacific (DKAP) project to ensure age-appropriate, regionally relevant content [31].

#### Coping strategies subscale

**Fig. 1:**
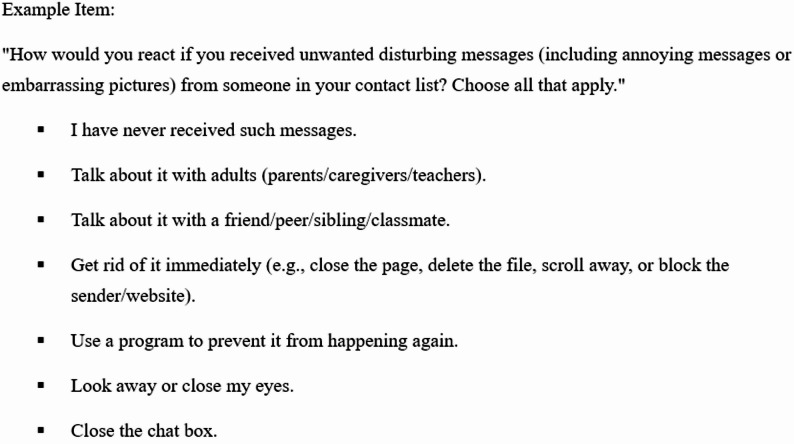
Example item of coping strategies subscale

This subscale evaluates three commonly reported coping families [6, 21]: productive coping (problem-solving and protective actions), reference to others (help-seeking from adults/peers), and non-productive coping (avoidant/passive responses). Because coping is context-dependent, we embedded items in three prevalent scenarios for children and adolescents: (1) unwanted disturbing messages (2), personal-information leakage, and (3) cyberbullying. For each scenario, students selected all strategies they used (endorsed = 1; not endorsed = 0). Skip logic routed students who had not experienced a scenario to the next section; those selecting “I don’t know what to do” were excluded from coping analyses. (see Fig. 1 as an example item).

The present Coping Strategies Subscale is grounded in a developmental-psychology perspective with the intention of reflecting age-related cognitive and socioemotional characteristics of children and adolescents. This subscale incorporates two socio-relational scenarios (unwanted disturbing messages; cyberbullying by friends) and one technical/abstract scenario (personal-information leakage), recognizing that exposure patterns and responses differ by risk type [32, 33]. The response options have been found to align with age-graded coping repertoires that have been documented in prior research. These repertoires include adult-referential and peer-referential help-seeking, proactive/technical actions (e.g., blocking, reporting, changing a password, or keeping evidence), and passive/avoidant responses (e.g., ignoring or closing the chat). These response options also align with established typologies of children’s online coping, as well as known primary–secondary differences in help-seeking and management strategies [19, 21, 33, 34]. Therefore, this design is consistent with the age-appropriateness of this scale for children and adolescents.

#### Recovery subscale

The Recovery Subscale assesses bouncing back (e.g., “I have learned to work together with classmates online”) and growth (e.g., “I am confident that I can quickly switch between face-to-face and online learning in the future”), each with four Likert-type items (1 = strongly disagree, 5 = strongly agree), where higher scores indicate stronger recovery or growth.

#### Theory-to-measure mapping and item generation

To ensure conceptual coherence, we mapped resilience-theory components to measurable facets and item formats in this DR scale. Please see Table 1.

**Table 1:** Mapping digital resilience with resilience in scale operationalization

Resilience Component	DR Application	Scale Operationalization
Risk-specific adaptation	Technology-mediated adversities differ in affordances and required responses	Three prevalent school-age scenarios: unwanted disturbing messages, personal information leakage, cyberbullying (scenario stems)
Coping as proximal mechanism	Mobilizing problem-focused and help-seeking behaviors vs. avoidant responses	Three coping families per scenario: productive coping (PC), reference to others (RtO), non-productive coping (non-PC)
Recovery and growth	Return to prior functioning and potential post-adversity gains	Two recovery factors with Likert items: bouncing back and growth

### Samples

We collected data from 2,014 primary school students and 6,014 secondary school students in 19 primary schools and 30 secondary schools in Hong Kong, of which a total of 1171 primary school students (58.14%) and 3944 secondary school students (65.58%) reported perceived online risks. As perceived online risks are antecedent, students who reported online risk experiences were included in this study. See Table 2 for detailed demographic information.

**Table 2:** Demographic information of student samples

School Level	Grade Level	Gender	*N*	%
Primary School	Primary 3–6	Male	565	11.00
		Female	582	11.40
		Missing	24	0.47
Secondary School	Junior Secondary (Secondary 1–3)	Male	1175	23.00
		Female	1557	30.40
		Missing	58	1.13
	Senior Secondary (Secondary 4–6)	Male	559	10.90
		Female	578	11.30
		Missing	9	0.18
		Male	4	0.08
	Missing	Female	1	0.02
		Missing	3	0.06

### Instruments

To examine the external validity of the Digital Resilience Scale, students also responded to a digital literacy assessment and a well-being scale.

#### Digital literacy

Student completed the 10-item Digital Literacy Assessment (DLA-short), a performance-based measure distilled from the validated long form (DLA-L) and aligned with the DigComp 2.1 framework [26, 35, 36]. The DLA-L has demonstrated robust psychometric properties, supporting a reliable unidimensional digital-literacy score in a sample of 4,016 students from 18 primary and 14 secondary schools in Hong Kong [35, 36]. The short form was constructed by selecting items that jointly optimized discrimination, difficulty, and content coverage. Items are multiple-choice with one to three keyed responses; scoring is dichotomous at the item level (1 = fully correct; 0 = otherwise).

#### Well-being

We assessed the subjective well-being using the 15-item Well-Being Profile—Short (WB-Pro-Short). Items (e.g., “I feel free to make my own choices”) were rated on a 9-point Likert scale (1 = completely disagree, 9 = completely agree) [37], with higher values indicating greater well-being.

### Data analysis

A series of psychometric analyses were conducted to address the research questions (RQs).

#### Examining the psychometric properties of DR scale

##### Examining the construct structure of digital resilience

For addressing RQ1, a separate confirmatory factor analysis (CFA) was conducted to examine the latent construct of recovery and learn from online risks All of the models were identified by setting the latent factor means at 0 and the latent factor variances at 1.

**Fig. 2:**
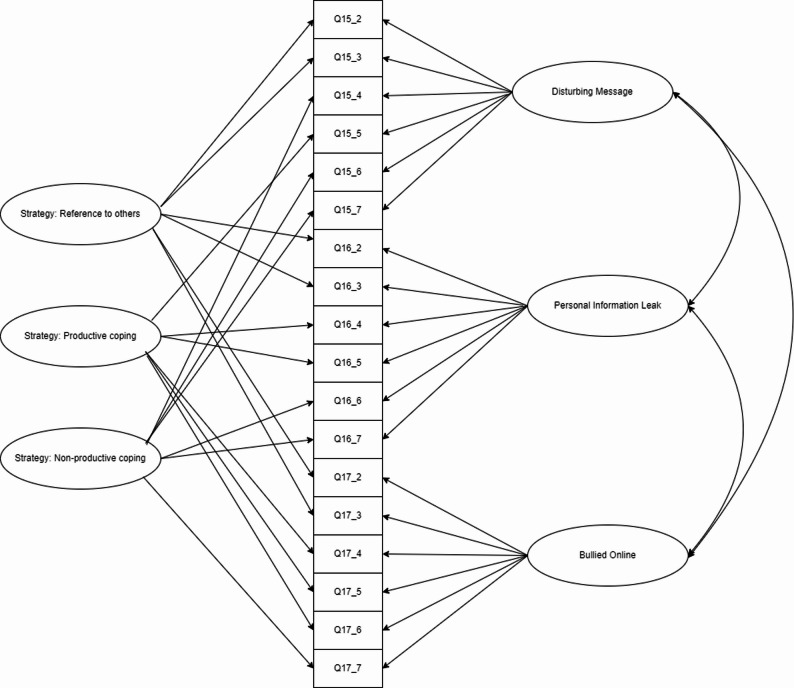
Diagram of two-tier full-information factor analysis model for students’ coping strategies

**Fig. 3:**
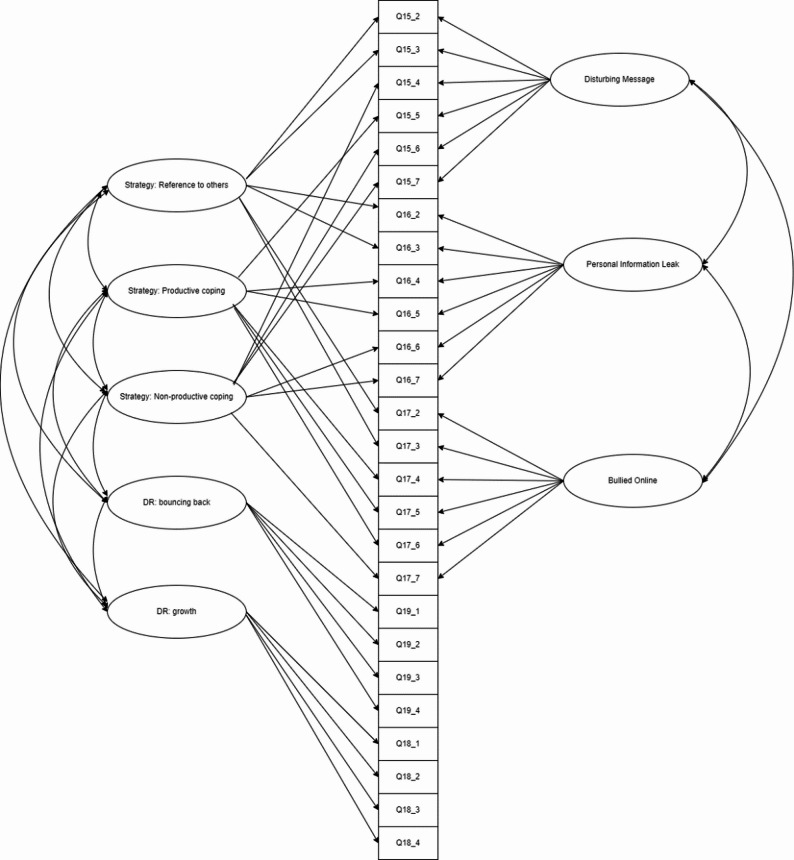
Diagram of full DR scale

To model dichotomous coping endorsements in our crossed design (three coping strategies within three risk scenarios), we used a two-tier full-information item factor analysis model [23]. TT-IFA generalizes standard multidimensional IRT, bifactor, and testlet response models by allowing multiple correlated primary factors (productive coping, reference-to-others, non-productive coping) while simultaneously capturing scenario-specific local dependence via orthogonal specific (“testlet”) factors—one per scenario—thereby avoiding bias from ignoring shared scenario context and wording effects (see Fig. 2). Concretely, each item loads on its target coping-strategy factor and on a single scenario factor; primary factors are freely correlated, whereas scenario factors are mutually orthogonal and orthogonal to the primaries. For identification and comparability, primary means were fixed to 0, primary variances to 1, and primary loadings were constrained equal across scenarios within each strategy (with scenario factors absorbing residual dependence); scenario-factor variances were fixed to 1. We estimated this confirmatory structure for categorical data using DWLS, which is appropriate with large samples and dichotomous indicators. This two-tier model matches our item design and offers a principled alternative to (a) multi-factor models that assume local independence and (b) the testlet model that would not represent three distinct coping dimensions. Last, a 5-correlated factor model (see Fig. 3) was conducted on the full DR scale to examine the psychometric properties of this DR scale.

##### Examining the measurement invariance (MI)

For addressing RQ2, MI was tested across gender and school levels (primary vs. secondary) to ensure the compatibility of the Digital Resilience Scale across gender and age groups. MI examines whether the psychometric properties of the observed indicators remain consistent across groups (e.g., gender, age) ([33], pp.3). In other words, it examines whether students’ responses to items are influenced by factors unrelated to the digital resilience, such as gender, age. Establishing measurement invariance is a prerequisite for meaningful factor mean comparisons and assessing associations across groups [24]. Given the documented gender and age differences in cyberbullying [32, 34, 38], examining MI in the Digital Resilience Scale is essential to ensure the compatibility of results across demographic groups.

Measurement invariance (MI) was evaluated in three steps: configural (same factor structure), metric (equal loadings), and scalar (equal intercepts). We fitted multiple-group CFA models (gender; school level) using MLR and compared nested models via likelihood-ratio tests [39]. Because full MI can be difficult to achieve in applied settings, we accepted partial invariance when theoretically justified [40, 41]. Nested model comparisons were performed via the likelihood ratio test ([39], pp206-286). As full MI is challenging to achieve in the applied research, partial invariance was accepted if necessary [40, 41].

##### Examining the reliability

McDonald’s [42] omega was used to calculate the reliability of DR scale, including students’ coping strategies, acceptance and growth). The reliability of a scale or a subscale is defined as the proportion of true-score variance to total observed variance [39]. The coefficient omega ranges from 0 to 1, and a higher value indicates higher reliability of the scale. The coefficient omega listed above assumes unidimensionality; therefore, it is suitable for the each sub-scale [43].

#### Examining the external validity of digital resilience

For addressing RQ3, given the established relationship between individual digital literacy, well-being and digital resilience in the literature [5, 44], we conducted a correlation analysis of students’ digital literacy, well-being and digital resilience (including the three coping strategies, bouncing back and growth) to examine the external validity evidence of this scale.

First, we calculated students’ DL and well-being scores. The students’ responses to DLA-short were calibrated by using a multiple-group unidimensional two-parameter logistic Item Response Theory (MG-2PL-IRT) model via maximum likelihood estimation (ML) and students’ DL score was estimated via Expected A Posteriori (EAP) estimation. A higher score indicates a higher overall level of DL. And the test reliability was assessed using EAP reliability estimates [45]. The reliability of DL scores was 0.91. Then, we used a single average score of the WB-Pro scale, ranging from 1 to 9, to represent students’ well-being, as suggested by the developers of the scale [37]. A higher mean score indicates a higher level of overall well-being. The scale showed excellent internal consistency (Cronbach’s $\:\alpha\:$ = 0.93).

Second, we estimated the correlations between individual digital literacy, well-being and digital resilience within a structural equation modelling (SEM) framework.

For all models, CFI and RMSEA were used to evaluate the model fit. Several studies have argued that the cut values of fit indices depend on sample size, loadings, and the desired power [46–48]. Therefore, we adopted RMSEA < 0.06 and CFI >0.95 as indicating a good fit and a CFI >0.90 and RMSEA < 0.08 as the minimum standard, as proposed by Hu and Bentler [45] and Rigdon [46]. All psychometric analyses were conducted in R using the “*lavaan*” package [49].

### Ethics approval

The project particulars and data collection methods were rigorously reviewed and approved by the Human Research Ethics Committee of [BLENDED FOR REVIEW] University.

## Results

### A multidimensional structure supported

First, each subscale of the DR scale was evaluated using a CFA separately, including three coping strategies, bouncing back, and growth. As shown in Table 3, all sub-scales demonstrated acceptable model fit, and all factors achieved acceptable reliability, with McDonald’s [42] omega values above 0.70, indicating each subscale exhibited sufficient reliability.

**Table 3:** Model fit summary

	Model Fit			Reliability
	CFI	RMSEA	95% CI of RMSEA	
Student-level factors				
Students’ coping strategy – RtO	1.00	< 0.001	(0.00, 0.06) (0.00, 0.07)	0.910
Students’ coping strategy – PC				0.810
Students’ coping strategy – non-PC				0.901
Students’ recovery – bouncing back	0.998	0.037	[0.022, 0.055]	0.854
Students’ recovery – growth	1.00	< 0.001	(0, 0.032]	0.764
Full model (5-correlated factor model)	0.967	0.046	[0.045,0.048]	-

*RtO* Reference to others, *PC* Productive coping, *non-PC* Non-productive coping

Second, a 5-correlated factor model was applied for the whole DR scale (See Fig. 2). Results showed that this model achieved sufficient model fit (CFI = 0.967, RMSEA = 0.046, 95% RMSEA CI = [0.045, 0.048]). The standardized factor loadings for all items were statistically significant (*p* < 0.01). Most items (25 out of 26) met or exceeded the recommended threshold of 0.50 [50], with factor loadings ranging from 0.55 to 0.84. Only one item (Q17_7 “Ignore them”) has a relatively low factor loading ($\:\lambda\:=0.31$); sensitivity analyses indicated that omitting it had a negligible effect on the non-PC factor (factor-score correlation with vs. without the item *r* = 0.993), so we retained it for content coverage and flagged it for future refinement. Detailed item-level results are provided in the additional file (see Additional file 1) to save the space. As shown in Table 4, correlations among the factors of digital resilience were estimated within an SEM framework. All factors were significantly interrelated. The three coping strategies showed moderate to high correlations with each other (coefficients ranging from 0.34 to 0.70). Meanwhile, the bouncing back and growth factors were highly correlated (*r* = 0.73) yet showed only moderate correlations with the coping strategies (coefficients ranging from 0.18 to 0.24, *p* < 0.001). These patterns suggest that while the factors are related, they capture distinct aspects of digital resilience, thereby supporting the scale’s hypothesized multidimensional structure.

**Table 4:** Correlations for investigated latent variables in digital resilience scale

	DR: RtO	DR: non-PC	DR: PC	DR: bouncing back	DR: growth
DR: RtO	1				
DR: non-PC	0.346	1			
DR: PC	0.566	0.703	1		
DR: bouncing back	0.242	0.074	0.185	1	
DR: growth	0.178	0.134	0.230	0.729	1

All correlation coefficients are significant at *p* = 0.001

*RtO* Reference to others, *PC* Productive coping, *non-PC* Non-productive coping

### Measurement invariance across gender and age groups

MI across gender and school level was assessed to ensure the fairness of the Digital Resilience Scale and the comparability of scores between different demographic groups. This analysis aimed to confirm that the scale measures digital resilience equivalently across genders and between primary and secondary school students.

As summarized in Table 5, the Likelihood Ratio Test (LRT) results supported partial MI across both gender and school level. Additionally, all models achieved acceptable model fit, indicating that constraining the factor loadings and intercepts did not compromise the fit of the models. These findings suggest that the scale functions equivalently across groups, allowing for valid comparisons of digital resilience between male and female students, as well as between students from primary and secondary schools.

**Table 5:** Measurement invariance results

		*N* of Parameters	$\:{\chi\:}^{2}$	df	*p*	CFI	RMSEA
MI across school levels							
DR: coping strategy	conf	84	2275.573	258	0.000	0.969	0.055
	metric	69	2290.092	273	0.000	0.969	0.054
	scalar	54	2696.316	288	0.000	0.963	0.057
	partial scalar	65	2298.719	277	0.000	0.969	0.053
DR: growth	conf	24	16.397	4	0.003	0.999	0.035
	metric	21	24.614	7	0.001	0.998	0.032
	partial metric	18	91.093	10	0.000	0.991	0.057
	partial scalar	20	27.586	8	0.001	0.998	0.031
DR: bouncing back	conf	26	1.393	2	0.498	1.000	0.000
	metric	23	25.679	5	0.000	0.998	0.040
	scalar	25	10.584	3	0.014	0.999	0.032
	partial scalar	25	10.584	3	0.014	0.999	0.032
MI across gender							
DR: coping strategy	conf	84	2401.726	258	0.000	0.966	0.058
	metric	69	2409.063	273	0.000	0.966	0.056
	scalar	65	2415.109	277	0.000	0.966	0.055
DR: growth	conf	24	19.597	4	0.001	0.998	0.040
	metric	21	21.021	7	0.004	0.998	0.028
	scalar	18	24.798	10	0.006	0.998	0.024
DR: bouncing back	conf	26	1.298	2	0.523	1.000	0.000
	metric	23	3.124	5	0.681	1.000	0.000
	scalar	20	26.071	8	0.001	0.998	0.030
	partial scalar	21	8.233	7	0.312	1.000	0.008

*MI* measurement invariance, *DR* digital resilience

Overall, the results demonstrate that the Digital Resilience Scale is suitable for assessing and comparing digital resilience across different gender and age groups, supporting its robustness and fairness in diverse educational contexts.

### Relationships between digital resilience, digital literacy, and well-being: external validity supported

As presented in Table 6, students’ DL exhibited significant positive correlations with two coping strategies (non-PC and PC), as well as with bouncing back and growth. Additionally, students’ well-being demonstrated significant positive relationships with two coping strategies (RtO and PC), bouncing back, and growth. Notably, DL was also positively correlated with well-being (*r* = 0.14, *p* < 0.001). Collectively, these findings underscore the positive relationships between some rather than all dimensions of digital resilience (DR) and students’ DL and well-being.

**Table 6:** Correlations among digital resilience, digital literacy, and well-being

	1	2	3	4	5	6	7
1 Students’ coping strategy – RtO	1						
2 Students’ coping strategy – non-PC	0.342	1					
3 Students’ coping strategy – PC	0.563	0.703	1				
4 Students’ recovery – bouncing back	0.244	0.083	0.189	1			
5 Students’ recovery – growth	0.154	0.062	0.147	0.619	1		
6 Digital Literacy	*0.036*	0.213	0.244	0.118	0.254	1	
7 Well-being	0.176	*0.042*	0.135	0.659	0.524	0.114	1

Only the correlations (*Italic numbers*) between DL and RtO and WB and nonPC are not significant. Others are all significant at 0.001

*RtO* Reference to others, *PC* Productive coping, *non-PC* Non-productive coping

## Discussion

This study evaluated the psychometric properties of a newly developed, multidimensional digital resilience (DR) scale among primary- and secondary-school students.

### Multidimensional nature of digital resilience

This study acknowledges the complexity of DR as a multidimensional concept that includes coping strategies, bouncing back, and growth. By examining the correlations between the factors of DR, we found that most factors had small to moderate correlations, with the exception of a strong correlation between bouncing back and growth. These findings suggest that DR is not a singular construct but a collection of distinct aspects. This is a crucial insight as it highlights that DR involves more than just coping with online adversity; it also encompasses the potential for personal growth through digital experiences [5, 6].

Notably, we observed a moderate correlation (*r* = 0.73) between bouncing back and growth. Previous studies have defined bouncing back as returning to prior functioning after encountering negative online experiences [51, 52]. From a developmental psychology perspective, overcoming adversity is understood as a dynamic and continuous process, encompassing both recovery and eventual progress or growth [10, 12]. In the context of DR, this process includes not only the ability to recover from adverse online experiences but also the capacity to learn and grow from them. Thus, while bouncing back signifies a fundamental component of resilience, it is the subsequent learning and growth that reflect the broader, more comprehensive nature of DR. At the same time, it is important to recognize these two constructs as distinct yet interconnected. Not all individuals who recover from risky online experiences will necessarily achieve personal growth or derive meaningful learning from them. To cultivate both recovery and growth, support at multiple levels—including individual, familial, community, and societal—plays a critical role in enabling adolescents to thrive online and develop robust digital resilience [15].

### Measurement invariance of digital resilience across gender and age

The investigation of gender differences in students’ experiences of online risks (e.g., cyberbullying) has been a long-standing research interest [34, 38, 53]. Consequently, there is a need for further research exploring gender differences in how students cope with these online risks, which constitute a component of DR. It is therefore essential to ensure that this DR scale is measured in a way that is consistent across genders.

Also, most studies on cyber wellness focus on secondary school students; however, as the average age of internet access continues to decrease [54], younger children are increasingly engaging with digital devices, leading to a rise in cyberbullying incidents in this demographic [28, 29, 32–34]. Consequently, there is a growing need to develop scales that can be applied to both primary and secondary school students. Therefore, measurement invariance across gender and age groups is essential for making fair comparisons. This study demonstrated sufficient measurement invariance across these groups, indicating that this digital resilience scale can be used to compare the digital resilience of primary and secondary school students, as well as across genders.

### External validity evidence: the relationships among digital literacy, digital resilience, and well-being

As expected, positive relationships were observed between the two coping strategies—productive coping (PC) and reference to others (RtO)—as well as between bouncing back, growth, and well-being. More specifically, these findings align with previous research that identified PC and RtO as indicators of greater resilience [6, 55]. The use of PC strategies reflects a proactive mindset in adolescents, where they actively seek solutions to mitigate the harm from online risks rather than avoiding the issues [6, 56]. Similarly, RtO strategies, which involve seeking support from parents and friends, have been shown to be beneficial in coping with online risks and resolving problems [19, 21]. Bouncing back and growth represent individuals’ ability to return to pre-risk levels of functioning and to experience personal growth as a consequence [15, 48, 51, 57]. Thus, it is logical to observe significant positive relationships between digital resilience factors and well-being.

In contrast, non-PC strategies, which are more passive and avoidant, are ineffective in addressing problems and may result in adverse outcomes regarding online risks [28]. Consequently, it is reasonable to conclude that there is no significant correlation between non-PC and students’ well-being.

Moreover, this study found that digital literacy (DL) was positively related to non-PC, PC, bouncing back, and growth. DL is widely recognized as a critical skill in the 21 st century, encompassing the knowledge, skills, and attitudes necessary to navigate effectively in a digital society [21]. Scholars emphasize that DL involves not only technical skills, such as accessing, managing, and communicating information, but also attitudes and values related to the safe and appropriate use of digital technologies for various purposes [58, 59]. In this study, DL was measured based on the DigComp 2.1 framework, which includes five areas of competence: information and data literacy, communication and collaboration, digital content creation, safety, and problem-solving [21]. Theoretically, these competencies, such as identifying online risks, seeking help through communication, and protecting well-being in digital environments, are related to digital resilience. Thus, it was expected that DL would be positively related to PC, bouncing back, and growth. However, the positive correlation between DL and non-PC (*r* = 0.214, *p* < 0.001) was unexpected. One possible explanation is that the scale captured the usage of strategies rather than their effectiveness. Students with higher DL might be more flexible in trying a variety of coping strategies, including both productive and non-productive approaches [60]. Finally, RtO was not significantly correlated with DL, likely because RtO is more about seeking help and communication, which may be more influenced by interpersonal relationships (e.g., parent-child relationships) [60] than by individual competencies.

### Limitations

Several limitations should be acknowledged in this study. First, one item (Q17_7, ‘Ignore them’) exhibited a sub-threshold loading (λ = 0.31); although sensitivity analyses showed negligible influence on the non-PC factor (factor-score *r* = 0.993 with vs. without the item), we retained it for content coverage and flag it for future revision. Second, the validation process was conducted within a specific region, which may limit the generalizability of the findings to other contexts. Future research could address this by collecting validity evidence from different regions or countries to explore the extent to which the scale can be applied across diverse populations and settings. Third, the rapid advancement of technology presents another challenge. As new digital risks and corresponding coping strategies continue to emerge, the scale may become outdated if not regularly reviewed and revised. To maintain relevance and accuracy, periodic updates to the scale will be necessary to capture new dimensions of DR and ensure it reflects the evolving landscape of digital environments. Further longitudinal studies could also provide insight into how DR evolves over time as technological developments unfold. Fourth, we did not administer an established unidimensional digital resilience scale, to reduce participant burden and survey fatigue. As a result, the current validation focuses on structural evidence and external associations (digital literacy, well-being) rather than direct convergent validity with an existing DR measure. Future studies should incorporate a brief, widely used unidimensional DR scale to provide criterion evidence and to evaluate the incremental validity of the present multidimensional instrument. In addition, we observed a moderate–high correlation (*r* = 0.73), suggesting overlapping variance while preserving discriminability; thus, we modelled them as distinct, correlated factors under recovery. A key limitation is that the current evidence is based on retrospective self-reports from a single source. This means that the temporal ordering of recovery processes cannot be established, and there is a potential threat of common method bias. To further clarify the relationship between bouncing back and growth and avoid potential common method bias, future studies should employ longitudinal or ecological momentary assessment and multi-informant designs to capture within-person dynamics. Last but not least, the present validation was conducted in Hong Kong, which may limit generalizability to other cultural–educational contexts. To address this, we have sought funding to implement the scale in Singapore and are engaging collaborators in additional regions. Our cross-cultural program will include cultural/linguistic adaptation (expert review), pilot testing, and a stratified field study, followed by measurement invariance (configural/metric/scalar) and differential item functioning analyses. We will also examine convergent evidence with a brief unidimensional DR measure and criterion associations (e.g., digital literacy, well-being) across contexts. These steps will provide stronger evidence for the use of the DR scale in a wider range of cultural settings.

## Conclusion

To conclude, this study developed a multidimensional digital resilience scale grounded in the Digital Resilience Framework, incorporating coping strategies, bouncing back, and growth. The scale was validated in a large sample of primary and secondary school students in Hong Kong, demonstrating the multidimensional nature of digital resilience, sufficient reliability, measurement invariance across genders and age groups, and expected external validity through positive correlations with students’ digital literacy and well-being. Together, these findings provide evidence that the scale is a psychometrically sound tool for assessing digital resilience in primary and secondary school students. This study contributes to both the scientific understanding of the multidimensionality of digital resilience and offers practical implications for measuring digital resilience in children and adolescent populations.

## Supplementary Information

Supplementary Material 1.

## Data Availability

All materials will be made available on request.

## Abbreviations

DR: Digital Resilience
DL: Digital Literacy
MI: Measurement Invariance
CFI: Comparative Fit Index
RMSEA: Root Mean Square Error of Approximation
RtO: Reference to others
PC: Productive coping
non-PC: Non-productive coping
