# Supplementary material for: Development and validation of digital resilience scale for primary and secondary school students
Source: BMC Psychol. 2025 Nov 25;13:1412. doi: 10.1186/s40359-025-03739-0 (PMC12750681; doi:10.1186/s40359-025-03739-0)
Supplement: Supplementary file 1 — Supplementary Material 1. [file 40359_2025_3739_MOESM1_ESM.docx]

Appendix

**Table A1**

Digital Resilience Scale

| **Coping strategy** | | |
| --- | --- | --- |
|  |  | How will you react when you receive unwanted disturbing messages (including annoying messages or embarrassing pictures from someone) from your contact list? Choose all that apply.  0=No  1=Yes |
| Q15 | 1 | I never received these messages. |
| Q15 | 2 | Talk about it with adults (parents/caregivers/teachers) |
| Q15 | 3 | Talk about it with a friend/peer/sibling/classmate |
| Q15 | 4 | Get rid of it immediately (by closing the page, deleting the file, scrolling away, or block the webpage or website) |
| Q15 | 5 | Use a program that prevents it from happening again |
| Q15 | 6 | Look away or close my eyes |
| Q15 | 7 | Close the chat box |
| Q15 | 8 | Don’t know what to do |
| Q16 |  | What will you do when you find that your personal information is obtained by others without your permission online? Choose all that apply.  0=No  1=Yes |
| Q16 | 1 | I never experienced this. |
| Q16 | 2 | Ask adults (parents/caregivers/teachers) to help |
| Q16 | 3 | Ask peers (sibilings/classmates/friends) to help |
| Q16 | 4 | Change password |
| Q16 | 5 | Use a report button |
| Q16 | 6 | Look away or close my eyes |
| Q16 | 7 | Close the chat box |
| Q16 | 8 | Don’t know what to do |
| Q17 |  | How will you react when you are bullied online by friends? Choose all that apply.  0=No  1=Yes |
| Q17 | 1 | I never experienced this. |
| Q17 | 2 | Talk with adults(tparents/caregivers/teachers) about what to do |
| Q17 | 3 | Talk with peers(sibilings/classmates/friends) about what to do |
| Q17 | 4 | Ask the persons to stop sending annoying messages or pictures |
| Q17 | 5 | Keep the evidence of bullying (e.g., screen shot) |
| Q17 | 6 | Delete or block the contact |
| Q17 | 7 | Ignore them |
| Q17 | 8 | Don’t know what to do |
|  | | |
| **Growth** | | |
| Q18 |  | With increasing experience of being online in the past 2 years (2020-2022), to what extent do you agree to the following statements?  5-point scale (1 = Strongly disagree, 5 = Strongly agree). |
| Q18 | 1 | I can quickly adapt to learning online at home if school lessons were to be suspended again. |
| Q18 | 2 | I can quickly adapt to communicating with my friends in-person when I need to. |
| Q18 | 3 | I am confident that I can quickly switch between face-to-face and online learning in the future. |
| Q18 | 4 | I am confident that I will not be spending too much time on social media. |
| **Bouncing Back** | | |
| Q19 |  | With increasing experience of homeschooling in the past 2 years (2020-2022), to what extent do you agree to the following statements?  5-point scale (1 = Strongly disagree, 5 = Strongly agree). |
| Q19 | 1 | I have learnt to work together with classmates online. |
| Q19 | 2 | I have learnt to communicate with my teachers online. |
| Q19 | 3 | I have set a clear routine when schools were suspended. |
| Q19 | 4 | I have learnt to control the amount of time I am spending online in social media. |

**Items of DR scale**

**Table A2**

Summary of factor loadings of DR scale

| Factor | Item | Std $\lambda$ | Std $\lambda$ SE | Std T$h$ | Std T$h$ SE | Std $\epsilon$ | Std $\epsilon$ SE |
| --- | --- | --- | --- | --- | --- | --- | --- |
| Students’ coping strategy – RtO | Q15_2 | 0.59 | 0.02 | 0.69 | 0.02 | 0.45 | 0.03 |
|  | Q15_3 | 0.62 | 0.02 | 0.60 | 0.02 | 0.37 | 0.03 |
|  | Q16_2 | 0.72 | 0.02 | 0.52 | 0.02 | 0.32 | 0.02 |
|  | Q16_3 | 0.80 | 0.02 | 0.75 | 0.02 | 0.13 | 0.03 |
|  | Q17_2 | 0.69 | 0.02 | 0.66 | 0.02 | 0.30 | 0.02 |
|  | Q17_3 | 0.75 | 0.02 | 0.72 | 0.02 | 0.14 | 0.02 |
|  |  |  |  |  |  |  |  |
| Students’ coping strategy – non-PC | Q15_4 | 0.66 | 0.02 | -0.02 | 0.02 | 0.29 | 0.03 |
|  | Q15_6 | 0.55 | 0.02 | 1.01 | 0.02 | 0.51 | 0.03 |
|  | Q15_7 | 0.58 | 0.02 | 0.25 | 0.02 | 0.46 | 0.02 |
|  | Q16_6 | 0.67 | 0.03 | 1.60 | 0.03 | 0.41 | 0.05 |
|  | Q16_7 | 0.71 | 0.02 | 0.89 | 0.02 | 0.34 | 0.03 |
|  | Q17_7 | 0.31 | 0.02 | 0.77 | 0.02 | 0.85 | 0.02 |
|  |  |  |  |  |  |  |  |
| Students’ coping strategy – PC | Q15_5 | 0.62 | 0.02 | 0.67 | 0.02 | 0.38 | 0.03 |
|  | Q16_4 | 0.74 | 0.01 | 0.03 | 0.02 | 0.29 | 0.02 |
|  | Q16_5 | 0.76 | 0.01 | 0.11 | 0.02 | 0.23 | 0.02 |
|  | Q17_4 | 0.71 | 0.01 | 0.62 | 0.02 | 0.25 | 0.02 |
|  | Q17_5 | 0.71 | 0.01 | 0.24 | 0.02 | 0.23 | 0.02 |
|  | Q17_6 | 0.62 | 0.02 | 0.50 | 0.02 | 0.42 | 0.02 |
|  |  | Std $\lambda$ | Std $\lambda$ SE | Std $Int$ | Std $Int$ SE | Std $\epsilon$ | Std $\epsilon$ SE |
| Students’ recovery – growth | Q18_1 | 0.76 | 0.01 | 3.31 | 0.03 | 0.49 | 0.01 |
|  | Q18_2 | 0.78 | 0.01 | 3.74 | 0.03 | 0.38 | 0.01 |
|  | Q18_3 | 0.80 | 0.01 | 3.53 | 0.03 | 0.33 | 0.01 |
|  | Q18_4 | 0.64 | 0.01 | 3.63 | 0.03 | 0.45 | 0.01 |
|  |  |  |  |  |  |  |  |
| Students’ recovery – bouncing back | Q19_1 | 0.80 | 0.01 | 3.65 | 0.03 | 0.36 | 0.01 |
|  | Q19_2 | 0.84 | 0.01 | 3.86 | 0.03 | 0.30 | 0.01 |
|  | Q19_3 | 0.67 | 0.01 | 3.22 | 0.03 | 0.55 | 0.01 |
|  | Q19_4 | 0.64 | 0.01 | 3.17 | 0.03 | 0.60 | 0.01 |

Note. Std = standardized; λ= factor loading; Th = threshold ; ϵ = residual; Int = intercept RtO = Reference to others; PC = Productive coping; non-PC = Non-productive coping
